# Metabolomic Evaluation of Air Pollution-related Bone Damage and Potential Mediation

**DOI:** 10.21203/rs.3.rs-2652887/v1

**Published:** 2023-03-28

**Authors:** Diddier Prada, Kathryn Rexrode, Vrinda Kalia, Charles Kooperberg, Alexander Reiner, Raji Balasubramanian, Hui-Chen Wu, Gary Miller, Iuliana lonita-Laza, Carolyn Crandall, David Cantu-de-Leon, Duanping Liao, Jeff Yanosky, James Stewart, Eric Whitsel, Andrea Baccarelli

**Affiliations:** Columbia University; Brigham and Women’s Hospital, Harvard Medical School; Columbia University; Fred Hutchinson Cancer Center; Fred Hutchinson Cancer Research Center; Brigham and Women’s Hospital; Mailman School of Public Health; Columbia University; Mailman School of Public Health; David Geffen School of Medicine at University of California; Instituto Nacional de Cancerologia; Pennsylvania State University College of Medicine; Pennsylvania State University College of Medicine; University of North Carolina; University of North Carolina; Mailman School of Public Health

## Abstract

Ambient air pollution has been associated with bone damage. However, no studies have evaluated the metabolomic response to air pollutants and its potential influence on bone health in postmenopausal women. We analyzed data from WHI participants with plasma samples. Whole-body, total hip, femoral neck, and spine BMD at enrollment and follow-up (Y1, Y3, Y6). Daily particulate matter NO, NO_2_, PM_10_ and SO_2_ were averaged over 1-, 3-, and 5-year periods before metabolomic assessments. Statistical analyses included multivariable-adjusted linear mixed models, pathways analyses, and mediation modeling. NO, NO_2_, and SO_2_, but not PM_10_, were associated with taurine, inosine, and C38:4 phosphatidylethanolamine (PE), at all averaging periods. We found a partial mediation of C38:4 PE in the association between 1-year average NO and lumbar spine BMD (p-value: 0.032). This is the first study suggesting that a PE may partially mediate air pollution-related bone damage in postmenopausal women.

## Introduction

Osteoporosis, a condition that weakens bones, making them more susceptible to sudden and resulting in bone fractures, occurs especially during aging and remains as a leading cause of disability, particularly in women, reducing independence and quality of life,^1,2^ and increasing morbidity and mortality in older individuals.^3,4^ About 2.1 million bone fractures are reported each year in the US, resulting in up to $20.3 billion in annual direct health costs, which could be dramatically reduced through early detection and effective preventive strategies.^5^ Risk factors for osteoporosis and bone fractures during aging include smoking, family history, low body mass index, long-term use of corticosteroids, anorexia/bulimia, heavy drinking, and long-term inactivity,^6^ but osteoporosis impacts women more than men, with 80% of the estimated 10 million Americans with osteoporosis being women.^7^

The postmenopausal period is a critical stage that enormously contributes to osteoporosis and fracture risk. Postmenopausal women have the highest fracture rates of any stage of lifespan.^8^ The absolute risk of fractures is much higher among postmenopausal women than pre- and perimenopausal women. Also, one in two women over 50 will experience a bone fracture because of osteoporosis.^9^ Therefore, studying risk factors for fracture postmenopausal women, such as factors that may have deleterious effects on bone density, can identify those at higher risk of bone fractures and allow us to target such individuals for early intervention strategies.

Recent studies from our group and others have revealed that long-term air pollution exposure reduces bone mineral density (BMD) and may increase bone fracture risk in later life.^10^ Our findings have also been now confirmed in multiple human studies^11–16^ and are supported by animal studies.^17^ We have also confirmed the effect of exposure to several air pollutants on reduced BMD and fractures in postmenopausal women.^18^ Recent human studies show that even low concentrations of ambient air pollution can affect the levels of several metabolites^19,20^ that affect bone-related metabolites such as α- tocopherol^21,22^ and glycine.^23,24^ Together, these findings suggest that ambient air pollution may “silently” increase bone damage many years before bone fractures occur. However, data on the relationship between air pollution, metabolomic profiles, and bone damage is unavailable, particularly among postmenopausal women. Here, we used metabolomics data available to identify novel metabolomic signatures of air pollution, estimate their association with BMD, and evaluate metabolomic mediation of the air pollution-bone damage association among participants in the Women’s Health Initiative (WHI).

## Results

### Characteristics of the participants

**Figure 1:**
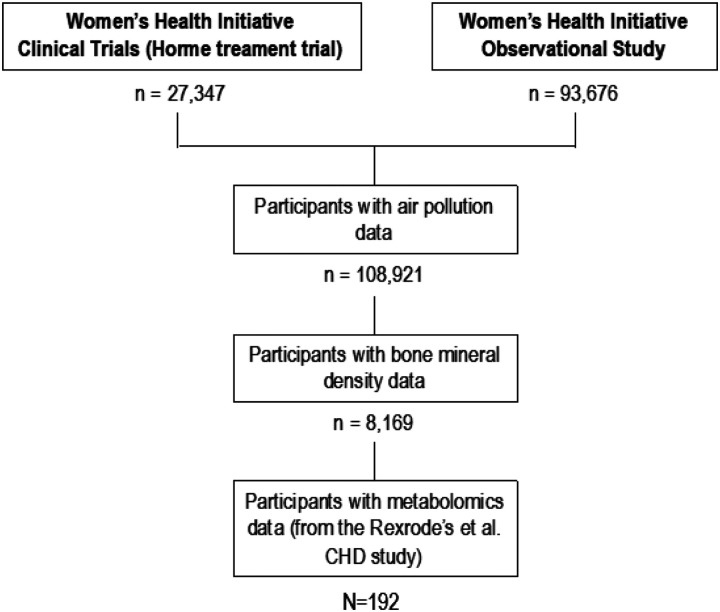
STROBE flow diagram for the analytic sample (i.e., the result of the serial application of exclusions) used in the current study. CHD: Coronary Heart Disease.

After the serial application of exclusions (Fig. 1), we obtained information on both air pollution and metabolomic assessment in 192 participants (N = 278 observations). Characteristics of these women at enrollment, by age group, are reported in **Supp. Table 1**. On average, WHI participants were aged 66.62 years (standard deviation [SD]: 6.83 years). Most participants were White (70.37% of those aged < 59 years; 71.71% of those 60–69 years; and 80.30% of those ≥ 70 years). The most common educational level was college or vocational (51.85% of those aged < 59 years; 41.41% of those 60–69 years; and 36.36% of those ≥ 70 years) and had a modest income (< $49,999/year); 97 (50.52%) participants were CHD cases and 95 (49.48%) controls.

### Metabolites and pathways associated with NO

**Figure 2:**
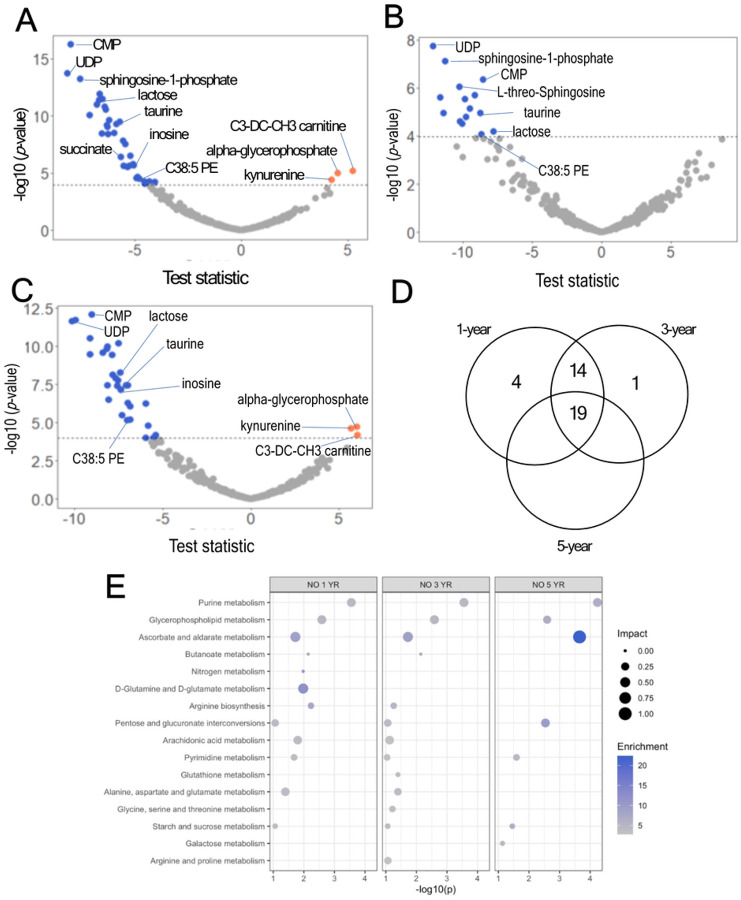
Associations of nitrogen oxide (NO) on the metabolomic response in postmenopausal women. **A-C.** Volcano plots for the association between 1-year (A), 3-year (B), and 5-year (C) average NO exposure before plasma assessment of plasma metabolites in Women’s Health Initiative participants (N=278 observations). **D.** Venn diagram showing shared metabolites between averaging periods. **E.** Significant metabolic pathways affected by nitrogen oxide (NO), 1-year (left), 3-year (center), and 5-year (right) average before plasma assessment (dotted line represents p < 0.05).

After adjustment for multiple hypotheses (i.e., Bonferroni), we found that NO exposure at the three averaging periods evaluated (1-, 3-, and 5-year average before plasma sampling) was associated with several metabolites, often showing a negative association, even after adjusting for multiple comparisons (Bonferroni adjusted p-value < 0.05). At the 1-year average window, NO was significantly associated with 37 metabolites, including mostly negative associations of cytidine monophosphate (CMP), sphingosine-1-phosphate, lactose, taurine, C38:5 phosphatidylethanolamine (PE), uracil-diphosphate (UDP) glucose, hexose monophosphate, 2-phosphoglycerate, uracil-monophosphate (UMP), and UDP-galactose. Positive associations were observed for alpha-glycerophosphate, homoarginine, kynurenine, and C38:5 PE, among others (Fig. 2, **Panel A**). A complete list of metabolites, estimates, and p-values (raw and adjusted) for the 1-year time window is shown in **Supplementary Table 2**. At the 3-year average window, NO was negatively and significantly associated with 34 metabolites, including UDP, sphingosine-1-phosphate, CMP, taurine, lactose, C38:4 PE, and C38:5 PE, among others. Positive associations were not observed for this time window (Fig. 2, **Panel B**). A complete list of metabolites, estimates, and p-values (raw and adjusted) for the 3-year time window is shown in **Supplementary Table 3**. At the 5-year average window, NO_x_ was significantly associated with 19 metabolites, with negative associations for CMP, UDP, lactose, taurine, sphingosine-1-phosphate, L-threo-sphingosine, UMP, 2-phosphoglycerate, guanosine diphosphate (GDP), UDP-galactose/UDP-glucose, ADP, C38:5 PE, and niacinamide, among others. Positive associations were observed for alpha-glycerophsophate, kynurenine, malonylcarnitine (C3-DC-CH3), and carnitine (Fig. 2, **Panel C**). A full list of metabolites, estimates, and p-values (raw and adjusted) for the 1-year time window is shown in **Supplementary Table 4**. We found 19 shared metabolites between the three averaging periods evaluated, including 2-phosphoglycerate, alpha-glycerophosphate, C38:5 PE, alphaglycerophosphocholine, CMP, and UMP (Fig. 2, **panel D**). For the one-year average, metabolic pathway enrichment analysis with Metaboanalyst revealed several altered metabolic pathways (Fisher exact test p-value < 0.05), including purine, glycerophospholipid, as well as ascorbate and aldarate metabolism, among others (Fig. 2, **panel E, left**). For the 3-year average, metabolic pathway enrichment analysis revealed alterations in several pathways, including purine, glycerophospholipid, as well as ascorbate and aldarate metabolism, among others (Fig. 2, **panel E, center**). For the 5-year average, metabolic pathway enrichment analysis revealed alterations in purine, ascorbate and aldarate metabolism, and glycerophospholipid metabolism, among others (Fig. 2, **panel E, right**).

#### Metabolites and pathways associated with NO_2_

**Figure 3:**
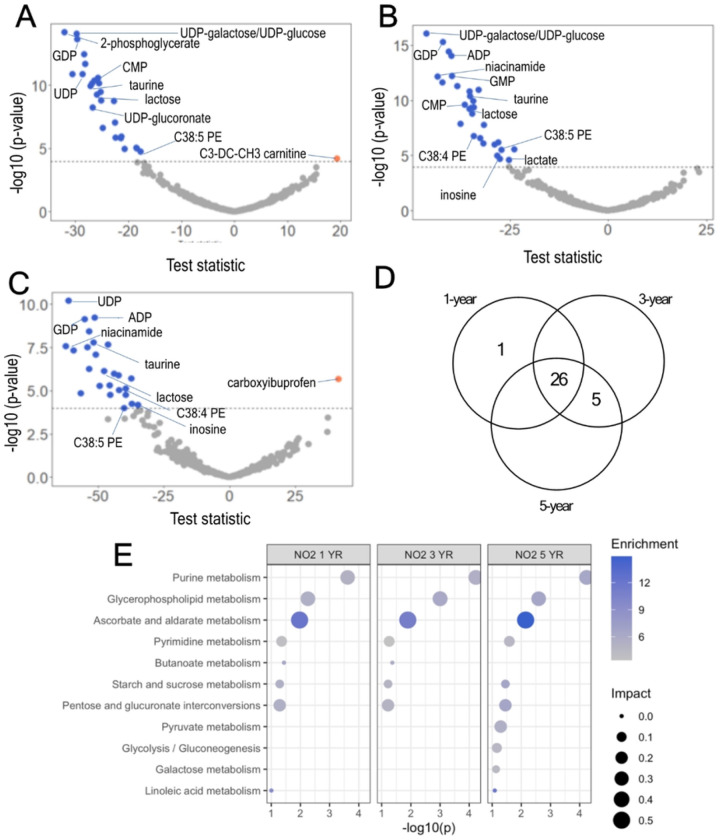
Associations of nitrogen dioxide (NO_2_) on the metabolomic response in postmenopausal women. **A-C.** Volcano plots for the association between 1-year (A), 3-year (B), and 5-year (C) average NO_2_ exposure before plasma assessment of plasma metabolites in Women’s Health Initiative participants (N=278 observations). **D.** Venn diagram showing shared metabolites between averaging periods. **E.** Significant metabolic pathways affected by nitrogen dioxide (NO_2_), 1-year (left), 3-year (center), and 5-year (right) average before plasma assessment (dotted line represents p < 0.05).

After adjustment for multiple hypotheses (i.e., Bonferroni), we found that NO_2_ exposure, at the three different averaging periods (1-, 3-, and 5-year average before plasma sampling), was associated with several metabolites, often showing a negative association after adjusting for multiple comparisons (Bonferroni < 0.05). At the 1-year average window, NO_2_ was significantly associated with 27 metabolites, including 2-phosphoglycerate, UDP-galactose/UDP-glucose, GDP, ADP, GMP, UDP, taurine, lactose, and niacinamide, among others (Fig. 3, **panel A**). A complete list of metabolites, estimates, and p-values (raw and adjusted) for the 1-year time window is shown in **Supplementary Table 5**. At the 3-year average window, NO_2_ was significantly associated with 31 metabolites, including 2-phosphoglycerate, GDP, UDP-galactose/UDP-glucose, ADP, niacinamide, GMP, UDP, hexose monophosphate, AMP, UMP, C38:4 PE, and taurine, among others (Fig. 3, **panel B**). A full list of metabolites, estimates, and p-values (raw and adjusted) for the 3-year time window is shown in **Supplementary Table 6**. At the 5-year average window, NO_2_ was also significantly associated with 28 metabolites, including UDP, ADP, GDP, L-threo-Sphingosine, hexose monophosphate, UDP-galactose/UDP-glucose, C38:4 PE, and taurine, among others (Fig. 3, **panel C**). We found 26 shared metabolites between the three averaging periods evaluated, including 2-phosphoglycerate, 6-phosphogluconate, adenosine, ADP, C38:4 PE, lactose, niacinamide, sucrose, taurine, and others (Fig.3, **panel D**). A full list of metabolites, estimates, and p-values (raw and adjusted) for the 5-year time window is shown in **Supplementary Table 7**. Metabolic pathway enrichment analysis revealed similar alterations for the three averaging periods evaluated, including purine metabolism, glycerophospholipid metabolism, ascorbate and aldarate metabolism, pyrimidine metabolism, butanoate metabolism, starch and sucrose metabolism, pentose and glucuronate interconversions, pyruvate metabolism, and others, which were similar between the three averaging periods evaluated (Fig. 3, **panel E**).

### Metabolites and pathways associated with SO_2_

After adjustment for multiple hypotheses (i.e., Bonferroni), at the 1-year average window, SO_2_ was not significantly associated with any of the metabolites evaluated (**Supplementary Fig. 1, panel A**). The full list of metabolites, estimates, and p-values (raw and adjusted) for the 1-year time window is shown in **Supplementary Table 8**. However, at the 3-year average window and after adjustment for multiple hypotheses (i.e., Bonferroni), SO_2_ was significantly associated with eight metabolites, including GDP, 6-phosphogluconate, ADP, GMP, adenosine, hexose diphosphate, UDP-galactose/UDP-glucose, and AMP (**Supplementary Fig. 1, panel B**). A full list of metabolites, estimates, and p-values (raw and adjusted) for the 3-year time window is shown in **Supplementary Table 9**. At the 5-year average window, SO_2_ was significantly associated with only three metabolites, including taurine, ADP, and GMP (**Supplementary Fig. 1, panel C**). We found three shared metabolites between the 3- and 5- averaging periods, including ADP, GMP, and taurine (**Supplementary Fig. 1, panel D**). A full list of metabolites, estimates, and p-values (raw and adjusted) for the 3-year time window is shown in the **Supplementary Table 10**. Metabolic pathway enrichment analysis revealed five altered metabolic pathways (Fisher exact test p-value < 0.05), including purine metabolism, ascorbate and aldarate metabolism, pentose and glucuronate interconversions, amino sugar and nucleotide sugar metabolism, and taurine and hypotaurine metabolism. Similar pathways were found associated with the 5-year average averaging periods (**Supplementary Fig. 1, panel E**).

### Metabolites and pathways associated with PM_10_

After adjustment for multiple hypotheses (i.e., Bonferroni), we found that PM_10_ at three different averaging periods (1-, 3-, and 5-year average before plasma sampling) was not significantly associated with any of the available metabolites (**Supplementary Fig. 2**). A full list of metabolites, estimates, and p-values (raw and adjusted) for the 1-, 3-, and 5-year averaging periods are shown in **Supplementary Table 11**–**13**.

### Air pollution-related metabolites also linked with BMD

**Figure 4:**
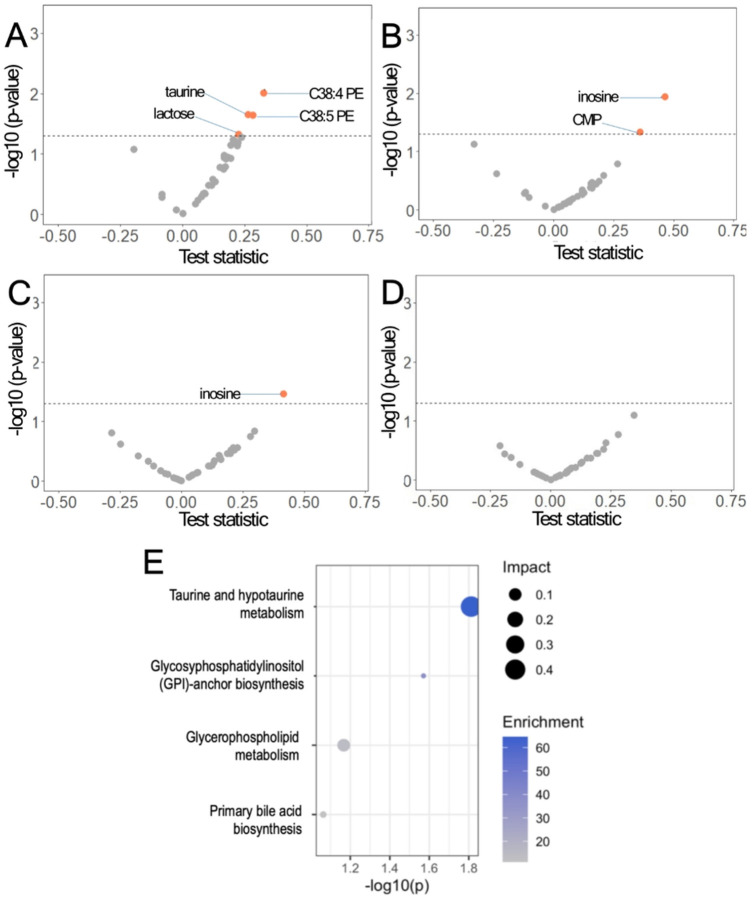
Volcano plot of the association between air pollution-related metabolites that were significant in the air pollution analysis in Women’s Health (N=285) at different anatomical sites. Lumbar Spine (A), Hip (B), Femoral Neck (C), and Total Body (D). E. Significant metabolic pathways associated with lumbar spine bone mineral density (inosine, C38:4 PE, taurine, and C38:5 PE) in Women’s Health Initiative participants (N=285, dotted line represents p < 0.05).

We tested whether air pollution-related metabolites were also linked with absolute bone mineral density (in g/cm^2^) and T-scores. In total, 36 metabolites were explored for this association. After adjustment for multiple hypotheses (i.e., Bonferroni), we found that C38:4PE, C38:5PE, taurine, and lactose were significantly associated with lumbar spine BMD (Fig. 4, **panel A**). For total hip BMD, only inosine and CMP were significantly associated at a Bonferroni-adjusted threshold (Fig. 4, **panel B**). For femoral neck BMD, only inosine was significantly associated (Fig. 4, **panel C**). For total body BMD, no air pollution-related metabolites were significantly associated (Fig. 4, **panel D**). Metabolic pathway enrichment analysis showed four altered metabolic pathways, including taurine and hypotaurine metabolism, glycosyphosphatidylinositol (GPI)-anchor biosynthesis, glycerophospholipid metabolism, and primary bile acid biosynthesis (Fig. 4, **panel E**). A full list of metabolites, estimates, standard errors, and p-values for the association with BMD are shown in **Supplementary Table 14**. Similar associations were observed for T-scores. A full list of metabolites, estimates, standard errors, and p-values for the association with T-scores are shown in **Supplementary Table 15**. Some changes were observed when sensitivity models explored the effects of smoking, alcohol consumption, physical activity, dietary modification or hormone therapy trials (**Supp. Figure 3**–**7**) on the association between air pollutants and BMD.

### Mediation modeling

We ran mediation modeling focusing on pollutants (i.e., nitrogen oxide), metabolites (i.e., inosine, taurine, C38:4 PE, C38:5 PE), and anatomical sites (i.e., lumbar spine BMD) with most substantial potential for mediation. Table 1 shows the mediation of the NO-lumbar spine BMD association by C38:4 PE. Following Baron and Kenny’s steps for mediation models,^25^ we determined the role of air pollution-related metabolites in lumbar spine BMD (b path). Although values of beta for the b path differ from the null for C38:4 PE, taurine, and lactose, no significant indirect effect was found for taurine or lactose. We found a partial mediation of the association between NO and lumbar spine BMD by C38:4 PE (Table 1) for the 1-year average time window, where C38:4 PE significantly accounted for 31% of the association (p-value: 0.032, raw p-value). No other metabolites showed statistical significance for mediation for any pollutant and averaging periods.

## Discussion

This study provides the first evidence of a metabolomic response mediating air pollution-related bone damage, particularly in a highly susceptible population: postmenopausal women. We identified an intense metabolomic response to two of the four pollutants evaluated, NO and NO_2_, but not SO_2_ or PM_10_. We also found that some of these metabolites were associated with BMD, particularly at the lumbar spine, total hip, and femoral neck. Remarkably, mediation models showed that C38:4 PE partially mediated the effect of NO on lumbar spine BMD. Our results suggest, for the first time, the involvement of critical bioactive phospholipids in air pollution-related bone damage.

In our study, we evaluated a large number of metabolites determined using an untargeted approach, including water-soluble metabolites (HILIC-pos and HILIC-neg columns), free fatty acids and bile acids (C18-neg columns), and polar and nonpolar plasma lipids (C18-pos). Initially, we observed extensive negative associations between NO exposure and multiple metabolites, indicating a potential vulnerability to long-term ambient air pollution. Metabolites commonly identified as associated with air pollutants included ADP, CMP, and taurine. Pathway analyses identified 16 metabolic pathways perturbed with long-term exposure to NO, including purine, glycerophospholipid, ascorbate and aldarate, and butanoate metabolism, among others. We also identified 11 metabolic pathways perturbed by long-term exposure to NO_2_, many shared with NO_x_, including purine, glycerophospholipid, ascorbate and aldarate, and butanoate metabolism, but not others, such as pyruvate metabolism, glycolysis/gluconeogenesis, and linoleic acid metabolism. SO_2_ exposure also showed an association with purine and ascorbate and aldarate metabolism, but also with amino sugar and nucleotide metabolism, as well as taurine and hypotaurine metabolism. Interestingly, no metabolomic response was observed in response to PM_10_.

These data suggest that air pollutants could trigger similar metabolomic pathways such as purine metabolism. Studies from Weichtal and colleagues have suggested that postmenopausal women are particularly susceptible to air pollution (i.e., NO), increasing their health risk of, for example, breast cancer.^26^ Ascorbate and aldarate metabolism has been reported as important for antioxidant activity in the human body, potentially acting as the first line of defense against inhaled pollutants.^27^ Our team has previously reported results from mixture analyses using Bayesian kernel machine regression models suggesting that bones in postmenopausal women may be particularly susceptible to air pollutants, especially NO.^18^ The current study offers the initial clues on the potential mechanisms underlying air pollution-related bone damage. To our knowledge, our results are the first to suggest strong and specific metabolomic signatures of long-term air pollution exposure in this susceptible population.

Arginine, one of the most versatile amino acids, is metabolically interconvertible with the amino acids proline and glutamate.^28^ It serves as a precursor for the synthesis of protein, nitric oxide, creatine, polyamines, agmatine, and urea.^28^ Similar to our findings for 1- and 3-year average NO exposure, a short-term (48 hour) exposure study by Liang and colleagues, identified arginine metabolism (as well as histidine, γ-linolenic acid, and hypoxanthine metabolism) as significantly associated with traffic indicators, including black carbon, carbon monoxide, nitrogen oxides, and fine particulate matter. Inflammatory and oxidative stress-related pathways, such as leukotriene and vitamin E metabolism, were also associated in that study, but not ours (except by aldarate metabolism that was not found in Liang’s study).^29^ Also, in a randomized study of clean air interventions, Li and colleagues found that arginine metabolism also was associated with PM_2.5_ exposure.^30^ These results suggest that arginine is consistently associated with air pollution and could be used as an exposure biomarker for biological effects of air pollutants.

On the other hand, Li and colleagues reported that purine metabolism was also linked to PM_2.5_ exposure, a report consistent with our finding of NO, NO_2_, and SO_2_ associations with purine metabolism at all averaging periods evaluated (except for 1-year average SO_2_).^30^ Purine metabolism plays an essential role in nucleic acid synthesis. Hu and colleagues showed that polycyclic aromatic hydrocarbon (PAH) and other air pollutant (e.g., PM_10_, NO_2_, and SO_2_) exposures were also linked with purine metabolism.^31^ Other studies on the effect of indoor air pollutants in older individuals with chronic obstructive pulmonary disease have also revealed a potential effect on purine metabolism.^32^ Other studies on the metabolomic response to air pollutants^33,34^ did not show metabolites to those observed by us, although the methodological design limits comparability between studies.

Air pollution-related metabolites, including taurine, lactose, C38:4 PE and C38:5 PE (two phosphatidylethanolamines), inosine, and CMP, were associated with bone mineral density, particularly at the lumbar spine, but also on femoral neck and total hip. Zhang and colleagues have previously identified 27 metabolites associated with femoral neck BMD.^35^ The glycine, serine, and threonine metabolism pathway (including four identified metabolites: creatine, dimethylglycine, glycine, and serine) were associated with BMD and improved the prediction and the classification of osteoporotic fracture risk beyond conventional risk factors.^35^ Early studies from You and colleagues using proton nuclear magnetic resonance spectroscopy suggested that, among postmenopausal women, elevated glutamine was significantly associated with low BMD and that elevated lactate, acetone, lipids, and very low-density lipoprotein were associated with high BMD.^36^ Miyamoto and colleagues, in unadjusted analyses, showed that postmenopausal women might show differential levels of metabolites such as pyruvate, lactate, succinate, urea, and creatine, among others in low-estrogen and low-BMD. Coincident with our results, they reported taurine as reduced in postmenopausal women with low BMD.^37^ Absence of common metabolites or pathways between these results and ours, except for taurine, may suggest that air pollution response is not related to age- or hormone-related bone damage. Taurine (2-aminoethane-1-sulfonic acid) is a sulfur-containing amino acid with a β-amino group and an acidic sulfonic group (R-SO3H) separated by two methylene (CH_2_) moieties. In humans, taurine plays a functional role in vital organs, such as the brain, eyes, kidneys, and heart. It performs several primary physiological functions, including osmotic regulation, and has antioxidant, antiapoptotic, and anti-inflammatory effects.^38^ Although taurine is not a structural component of proteins, it is metabolically involved in many processes that in fluence bone development and promote osteoblastogenesis. Studies in postmenopausal Brazilian women with osteopenia or osteoporosis have shown reduced taurine levels in plasma compared to healthy volunteers.^39^ Similar results were observed in White pre-menopausal women in the USA, Japanese women with low estradiol and BMD levels, and older Chinese adults with low BMD.^40^ Metabolomic signatures associated with age-related BMD reductions go further than our intended research. Further research about the potential implications of taurine in bone metabolism for air pollution-related bone mineral reductions is guaranteed.

Our results from mediation modeling suggest a critical role of C38:4 PE, a phosphatidylethanolamine, in the association between air pollution (i.e., NO) and bone damage. For many years, long-term exposures to air pollution have been linked to oxidative stress.^41^ Reactive oxygen species (ROS) include unstable shortlived molecules that contain oxygen (O_2_., H_2_O_2_, and OH^−^) and are highly reactive in cells.^42^ Superoxide anion may give rise to a variety of reactive carbonyl species called reactive aldehydes (RA). RAs can be more destructive than ROS, as their lives are longer (minutes to hours), and their structure allows them to migrate long distances and induce damage to cellular components, such as phospholipids, including phosphatidylethanolamines (PEs). PEs are considered the second most abundant phospholipid in mammalian cells and comprises about 15–25% of the total lipid in mammalian cells, after phosphatidylcholine.^43^ PE is enriched in the inner lea et of membranes, and it is especially abundant in the inner mitochondrial membrane.^43^ Due to its conical shape, PE modulates membrane curvature and lateral pressure^44,45^ and thus supports membrane fusion^46–48^ and the function of several membrane proteins.^49,50^ Oxidative damage by RAs on PE may induce PE adducts (e.g., HNE-Michael adducts, HNESchiff adducts, and ONE-Schiff adducts),^42^ potentially affecting cell membrane domains stability (lipid rafts), including bone cells. PE-derived adducts have been proposed as mediators of RA effects on membrane proteins.^42^ In fact, evidence suggests that PE added to metal implants increases mesenchymal stem cell osteoblastogenesis.^51^ Osteoclasts, one essential component of bone resorption, require PE for osteoclastogenesis.^52^ Studies from Irie and colleagues have found that immobilization of the cell surface PE blocked osteoclast fusion, suggesting a critical role of PE abundance and distribution for osteoclast generation.^52^ In this context, we hypothesize that air pollution-induced oxidative damage could be inducing RAs that interact with bone cells PE, particularly mononuclear pre-osteoclasts, affecting osteoclastogenesis and, therefore, bone resorption and bone physiology in general. A summary of this potential physiopathological mechanism is shown in **Supplementary Fig. 8**.

Like any other study, ours has some limitations. first, as this study was performed only in the US and included only menopausal women, these results may not be generalized to other populations. However, our population included White, Black, Hispanic, and women from other races/ethnicities, contributing to the potential generalizability of the results. Also, our and other studies have suggested the effect of air pollutants on men. Therefore, it is necessary to confirm whether this metabolomic response is sex-specific or if there is a common metabolomic response and mediation between men and women. Another limitation of our study was our inability to include PM_2.5_, which may also trigger bone damage.

Unfortunately, most air pollution predictions were made in the 1990s, and the US EPA Federal Reference Method network for PM_2.5_ was established in 1999,^53^ this fraction was not included in the analysis. Our results could also be influenced by self-correlation (e.g., NO and NO_2_ are pollutants with similar sources). However, these self-correlated pollutants exposure occur in similar contexts. On the other hand, our study has several strengths, including a well-validated metabolomics platform, detailed covariate information with a prospective design (baseline and year 1 of follow-up), and a robust methodology.

## Conclusions

This is the first study showing a strong metabolomic response to criteria air pollutants, with potential effects on bone mineral density, suggesting that PEs are critical mediators of air pollution-related damage in postmenopausal women. Studies that discover the potential implications of these metabolites and pathways on clinically relevant outcomes (i.e., bone fractures) are needed. Experimental intervention studies, increasing the levels of those metabolites negatively affected by air pollution and directly linked with bone health, are also encouraged by our results.

## Methods

### Population

The population included in this study was drawn from the Women’s Health Initiative – Observational Study (WHI-OS) and Clinical Trials (WHI-CT) of postmenopausal United States women who enrolled between 1993 and 1998.^54^ All participants provided written informed consent. In the original metabolomic study,^55^ 784 participants who developed CHD after the baseline examination (cases) were selected, and 787 participants who did not develop CHD were matched on 5-year age, race/ethnicity, hysterectomy status, and 2-year enrollment groups. From them, 140 participants with CHD and 138 controls were included in the current study based on the availability of air pollution and BMD data.

### Air pollution exposure data

WHI participant addresses from study initiation to date have been geocoded.^56,57^ Geocoded participant address-specific daily mean concentrations of PM_10_(μg/m^3^))between 1993 and 2012 have been spatially estimated using available US Environmental Protection Agency Air Quality System (AQS) data and national-scale, log-normal, ordinary kriging.^58–60^ Analogous participant address-specific daily mean concentrations of gaseous pollutants (nitrogen oxides [NO, [parts per million, ppm]; nitrogen dioxide [NO_2_,ppm]; and sulfur dioxide [SO_2_, ppm]) also have been estimated using the same methods. In addition, monthly mean geocoded participant-address specific concentrations of PM_10_ have been spatiotemporally estimated using generalized additive mixed models and geographic information system-based predictors. The pollutant-, duration- and model-specific estimates were averaged over one, three, and five years before (and ending on) dates of plasma metabolomic assessment. As most of the air pollution predictions were made in the 1990s and the US Environmental Protection Agency (EPA) Federal Reference Method network for particulate matter < 2.5 μm (PM_2.5_) was established in 1999,^53^ this particle fraction was not included in the analysis.

### Metabolomic data

Plasma samples were collected using EDTA tubes in the WHI at baseline and Y1 and processed immediately. All WHI specimens were stored in a − 70°C freezer within 2 hours of collection or stored at − 20°C for up to 2 days, then shipped on dry ice and stored at − 70°C until processing. The majority of the WHI samples had been thawed once before our study. Metabolomic measurements were made using four complementary liquid chromatography-tandem mass spectroscopy (LC-MS) methods resulting in 371 metabolites. For each liquid chromatography (LC) method ([HILIC]-positive, C8-positive, C18-negative), pooled plasma reference samples were included after every 20 samples, and results were standardized using the ratio of the value of the sample to the value of the nearest pooled reference multiplied by the median of all reference values for the metabolite. Other details about the metabolomic assessment of these samples are described elsewhere.^55^

### Bone mineral density assessments

The WHI Bone Density Substudy, in which BMD (g/cm^2^) was measured at enrollment, year 1, year 3, and year 6 clinic visits, included all participants at three clinical centers (Birmingham, AL; Pittsburgh, PA; and Tucson, AZ) chosen to maximize racial diversity; and a satellite clinic (Phoenix, AZ) (N = 11,020). Those participants without air pollution estimations were excluded. Therefore, for the current study, we analyzed data from the WHI - Clinical trial (CT) and WHI - Observational Study (OS) participants (n = 9,041 participants: n = 4,202 from the CT and n = 4,839 from the OS). Participants of this WHI BMD cohort underwent dual-energy x-ray absorptiometry (DXA) measurement using Hologic machines (QDR2000, 2000+, or 4500). Quality assurance methods included cross-clinic calibration phantoms and a review of a random sample of scans. When the Hologic QDR 2000 machines were upgraded to QDR 4500 machines, in vivo cross-calibration procedures were performed, and results were adjusted for these correction factors and for longitudinal changes in scanner performance. T-score was also evaluated as an outcome. The reference standard from which the T-score is calculated is the female, White, age 20–29 years, NHANES III database.^61^ We used measurements of BMD at the total hip, lumbar spine, and total body in g/cm^2^ from DXA scans performed on each participant at enrollment and the years 3 and 6 clinic visits.

## Statistical Analyses

### Relationship between air pollutants and circulating metabolites

We performed the necessary data preprocessing and quality control steps, such as checking for normality and the presence of outliers. In the case of non-normal distributions of continuous variables, data transformation (e.g., log transformation) was used. In the case of missing data, multiple imputation protocols were established to maintain statistical power. For the effect of air pollutants on the metabolome, we examined whether 1-, 3-, and 5-year mean pollutant (PM_10_, SO_2_, NO, and NO_2_) concentrations prior to the examination cycle (metabolomic assessment) were longitudinally associated with the relative abundance of the metabolites. We fit linear mixed-effects models for continuous outcomes and adjusted for potential confounders (i.e., age at each visit [years], body mass index [kg/m^2^], ethnicity [(White, Black, Hispanic, Others: American Indian/Alaska Native, and Asian/Pacific Islander)], education [High school or less; College or vocational school; Grad school or higher], and coronary heart disease [yes; no] while controlling for the repeated evaluation [baseline and visit 1]). For the linear mixed-effect models, we assumed the following model: Y_ij_ = α_0_ + β_1_Z_1i_ + β_2_X_2ij_ + b_0i_ + e_ij_, where Y_ij_ and X_ij_ represent the metabolomic levels and the potential confounders at the j^th^ measurement on the i^th^ subject, and Z_1i_ is the pollutant concentration for the i-th individual at time point j. b_0i_ and e_ij_ correspond to random effects and random error effects, respectively. We used Bonferroni correction for multiple testing (371 metabolites). Sensitivity models explored the effect of several covariates (smoking [yes/no], alcohol consumption [yes/no], physical activity [> 0.03 Metabolic equivalent hours/week], dietary modification [case/control], or hormone therapy trials [case/control]) on the association with BMD.

### Relationship between circulating metabolites and bone mineral density

Only air pollution-related metabolites were evaluated for associations with BMD using multivariable-adjusted linear mixed models. Models were adjusted for potential confounders (i.e., age, body mass index, ethnicity, education, and coronary heart disease [yes; no] while controlling for the repeated evaluation [baseline and visit 1]).

### Mediation analysis

We ran mediation models following Baron and Kenny’s steps.^25^ We assessed whether the effect of key air pollutants on target BMD anatomical sites was mediated by those metabolites associated with air pollutants and BMD using multiple mediator models (**Supp. Figure 9**). Coefficients were obtained from a linear regression analysis. Indirect effects were calculated using the product-of-coefficients method (a*b).^62^ Standard errors and confidence intervals for mediation analyses were calculated by bootstrapping (5000 samples).^63^ Outcome and mediating variables were adjusted for age (years), race/ethnicity (White vs. non-White), body mass index (kg/m^2^), and education level (primary vs. higher education). The direct effect (c’ path) did not have to be reduced to zero because an incomplete mediation of the effect was expected. Also, for significant mediating variables, the proportion mediated was calculated as an effect size measure ((a*b)/c).^64^ Because of the low number of metabolites included in the mediation models, no correction for multiple testing was included. We performed the analyses using R software (R Project for Statistical Computing, CRAN, The Comprehensive R Archive Network, Vienna) and the command mediate() in the ‘mediation’ package for bootstrapping. A Bonferroni-adjusted p-value < 0.05 identified results as statistically significant.

### Pathway Identification

To better understand the biological significance of the metabolites, we conducted pathway analysis using metabolites significantly associated (at p-value < 0.05) with air pollution and the top metabolites that have the greatest weighting on the significant independent component analysis-factor(s). We used the “Pathway Analysis” functionality in MetaboAnalyst 5.0, which accounts for both over-representation (i.e., how many significant metabolites fall within a given pathway) and pathway topology (i.e., how important those metabolites are to that pathway)^65^ using the Human Metabolome Database for metabolite IDs and KEGG databases for pathway mapping. We considered statistical significance for pathways at a nominal *p*-value ≤ 0.1 and considered additional noteworthy pathways if the impact score was ≤ 0.5 while the 0.1 ≥ nominal *p*-value < 0.3.

### Ethics Statement

We confirm that the use of human tissue samples (i.e., plasma) was performed in accordance with the Declaration of Helsinki. Written informed consent was obtained from all participants at randomization/enrollment. All protocols were approved by each of the 40 WHI centers, including the Fred Hutchinson Cancer Research Center (Seattle, WA), Oregon Health & Science University (Portland, OR); University of Nevada (Reno, NV); University of California, Davis (Davis, CA); Stanford University (Stanford, CA); Kaiser Foundation Research Institute (Oakland, CA); University of California, Los Angeles (Los Angeles, CA); University of California, Los Angeles (Torrance, CA); University of California, Irvine (Irvine, CA); University of California, San Diego (La Jolla, CA); University of Arizona (Tucson/Phoenix, AZ); University of Texas, San Antonio (San Antonio, TX); Baylor College of Medicine (Houston, TX); University of Miami (Miami, FL); University of Florida (Gainesville/Jacksonville, FL); Emory University (Atlanta, GA); University of Alabama at Birmingham (Birmingham, AL); University of Memphis (Memphis, TN); University of North Carolina, Chapel Hill (Chapel Hill, NC); Wake Forest University (Winston-Salem/Greensboro, NC); George Washington University (Washington, DC); MedStar Research Institute (Washington, DC); University of Cincinnati (Cincinnati, OH); University of Pittsburgh (Pittsburgh, PA); University of Medicine and Dentistry of New Jersey (Newark, NJ); Albert Einstein College of Medicine (Bronx, NY); State University of New York, Stony Brook (Stony Brook, NY); Memorial Hospital of Rhode Island (Pawtucket, RI); Brigham and Women’s Hospital (Boston, MA); University of Massachusetts (Worcester, MA); State University of New York, Buffalo (Buffalo, NY); Wayne State University (Detroit, MI); The Ohio State University (Columbus, OH); Rush University Medical Center (Chicago, IL); Northwestern University (Chicago/Evanston, IL); Medical College of Wisconsin (Milwaukee, WI); University of Wisconsin (Madison, WI); University of Iowa (Iowa City/Bettendorf, IA); University of Minnesota (Minneapolis, MN); University of Hawaii (Honolulu, HI).^66^

## Figures and Tables

**Table 1.** Metabolite mediation of the association between 1-year average nitrogen oxide (NO) concentration and lumbar spine bone mineral density in WHI participants.

	X → Y (c path)^a,h^									
	β	95% CI								
Crude analysis	−1.213	(−2.251, −0.174)								
	X → Y (c’ path) direct^b,h^		X → M (a path)^c,h^		M → Y (b path)^d,h^		Indirect effect (a*b)^e,g^		Proportion mediated^d,f^	
Metabolite	β	95% CI	β	95% CI	β	95% CI	β	95% CI	β	95% CI
C38: 4 PE	−0.836	(−1.915, 0.243)	−4.780	(−6.620, −2.939)	0.092	(0.029, 0.154)	−0.377	(−0.784, −0.040)	0.310	(0.023, 1.360)
C38: 5 PE	−1.067	(−2.123, −0.010)	−2.950	(−4.796, −1.103)	0.060	(−0.006, 0.126)	−0.146	(−0.412, 0.060)	0.120	(−0.070, 0.610)
Taurine	−0.829	(−1.965, 0.307)	−5.688	(−7.208, −4.167)	0.094	(0.019, 0.168)	−0.384	(−0.821, 0.040)	0.320	(−0.054, 1.500)
Lactose	−0.862	(−2.034, 0.310)	−6.673	(−8.211, −5.134)	0.083	(0.010, 0.155)	−0.351	(−0.861, 0.140)	0.290	(−0.169, 1.510)

a c path (total effect): The crude association between NO (1-year average) and bone damage.

b c’ path (direct effect): the association between NO (1-year average) and bone damage, adjusted for mediator (metabolite levels).

c a path: association between NO (1-year average) and metabolite levels.

d b path: association between metabolite levels and bone damage.

e Indirect effect (a*b): the indirect effect of the NO (1-year average) on bone damage through metabolite levels

f Proportion effect mediated ((a*b)/c): the proportion of the total effect mediated through metabolite levels.

g Confidence interval for indirect effects were calculated with bootstrapping (5000 samples).

h All analyses used linear regression models adjusted for age (as a continuous variable) race (White vs non-white). BMI (as continuous variable), and education level (primary vs. higher education).

## Data Availability

Metabolomics data are available from the corresponding author on reasonable request and after approval of WHI and datasets owners (Dr. Rexrode).
